# Active longevity and aging: dissecting the impacts of physical and sedentary behaviors on longevity and age acceleration

**DOI:** 10.1007/s11357-024-01329-3

**Published:** 2024-09-04

**Authors:** Ting Yu Lu, Jiao Wang, Chao Qiang Jiang, Ya Li Jin, Kar Keung Cheng, Tai Hing Lam, Wei Sen Zhang, Lin Xu

**Affiliations:** 1 School of Public Health, Sun Yat-Sen University, Guangzhou, 510080 China; 2 Guangzhou Twelfth People’s Hospital, Guangzhou, 510620 China; 3 School of Public Health, the University of Hong Kong, Hong Kong, China; 4 Institute of Applied Health Research, University of Birmingham, Birmingham, B15 2TT UK; 5 Greater Bay Area Public Health Research Collaboration, Guangzhou, China

**Keywords:** Physical activity, Sedentary behavior, Longevity, Age acceleration, Mendelian randomization, Mediation analysis

## Abstract

**Background:**

To examine the associations of physical activity (PA) and sedentary behavior (SB) with longevity and age acceleration (AA) using observational and Mendelian randomization (MR) studies, and quantify the mediating effects of lipids.

**Methods:**

In Guangzhou Biobank Cohort Study (GBCS), PA and SB were assessed by the Chinese Version of the International Physical Activity Questionnaire. Longevity was defined as participants whose age at follow-up or at death was at or above the 90th age percentile. AA was defined as the residual resulting from a linear model that regressed phenotypic age against chronological age. Linear regression and Poisson regression with robust error variance were used to assess the associations of total and specific PA in different intensities, and SB with AA and longevity, yielding βs or relative risks (RRs) and 95% confidence intervals (CIs). Two-sample MR was conducted to examine the causal effects. Mediation analysis was used to assess the mediating effects of lipids.

**Results:**

Of 20,924 participants aged 50 + years in GBCS, during an average follow-up of 15.0 years, compared with low PA, moderate and high PA were associated with higher likelihood of longevity (RR (95% CI): 1.56 (1.16, 2.11), 1.66 (1.24, 2.21), respectively), and also cross-sectionally associated with lower AA (β (95% CI): -1.43 (-2.41, -0.45), -2.09 (-3.06, -1.11) years, respectively). Higher levels of moderate PA (MPA) were associated with higher likelihood of longevity and lower AA, whereas vigorous PA (VPA) showed opposite effects. The association of PA with longevity observed in GBCS was mediated by low-density lipoprotein cholesterol (LDL-C) by 8.23% (95% CI: 3.58–39.61%), while the association with AA was mediated through LDL-C, triglycerides and total cholesterol by 5.13% (3.94–7.30%), 7.81% (5.98–11.17%), and 3.37% (2.59–4.80%), respectively. Additionally, in two-sample MR, SB was positively associated with AA (β (95% CI): 1.02 (0.67, 1.36) years).

**Conclusions:**

PA showed protective effects on longevity and AA, with the effects being partly mediated through lipids. Conversely, SB had a detrimental impact on AA. MPA was associated with higher likelihood of longevity and reduced AA, whereas VPA showed adverse effects. Our findings reinforce the recommendation of “sit less and move more” to promote healthy longevity, and highlight the potential risks associated with VPA in the elderly.

**Supplementary Information:**

The online version contains supplementary material available at 10.1007/s11357-024-01329-3.

## Introduction

With rapid development of economy, human life expectancy has dramatically increased [1]. Longer life expectancy has led to a rapidly aging population occurred [2], causing multiple challenges such as aging-related diseases and significant financial burdens. While longevity refers to the duration of life, aging encompasses physical, biological, and psychological changes, often measured in terms of biological age. Age acceleration (AA) indicates how quickly these changes are happening compared to chronological age [3].

Previous studies consistently showed that low levels of physical activity (PA, defined as any bodily movement produced by skeletal muscles that requires energy expenditure) and high levels of sedentary behavior (SB, defined as any period of low-energy expenditure while awake such as sitting, reclining or lying) were associated with higher risks of mortality and morbidity [4, 5]. However, evidence on the associations of PA, SB with longevity remained underexplored. Observational studies reporting the association of SB with longevity using a practical definition based on the age distribution of the study population are lacking. Regarding the association between PA and longevity, current observational studies using inconsistent single age threshold to define longevity showed various findings, with inverse U-shaped association [6], no association [7], or sex-stratified results [8] being reported. While much of the existing literature has focused on epigenetic markers of AA [9–17], the measurement of epigenetics is costly and not always feasible for clinical practice. Phenotypic age, as an alternative marker, is more accessible. We found two studies that examined the associations of PA, SB with phenotypic AA, with one showing a U-shaped association with accelerometer-measured PA intensity [18], while the other found a negative association with self-reported PA and a positive association with SB [19]. Furthermore, most studies have focused on total PA level rather than on frequencies and durations of PA in specific intensities, which could better guide the public in exercising [6–8, 18]. Older adults face a series of functional declines, including decreased muscle strength, balance, and cardiopulmonary function, which increase the risks of falls and cardiovascular disease (CVD) [20, 21]. Additionally, malnutrition is common among the elderly, potentially leading to insufficient energy reserves to sustain vigorous PA (VPA) [22]. Therefore, the impact of VPA may vary significantly from that of walking or moderate PA (MPA) in this population, thereby warranting further investigation for clarification.

Furthermore, residual confounding is a persistent challenge in traditional observational studies, precluding definitive causal conclusions [23]. The extended duration necessary to study outcomes like longevity and aging renders randomized controlled trial impractical. Mendelian randomization (MR), as an alternative design to infer causality, provides less biased estimates [23]. To date, only two MRs examined the associations of PA and SB with longevity and found no significant association [24, 25]. However, due to the use of a limited number of single-nucleotide polymorphisms (SNPs) (5 for PA and 4 for SB) [25], or the minor increment of the continuous PA variable per unit [24], all estimates in these two studies showed non-significant results with wide confidence intervals (CIs). Our search did not yield any MR studies examining the associations of PA and SB with phenotypic AA. Furthermore, lipids have been shown to be significantly affected by PA and SB [26], and PA was also recommended as a critical component of first-line treatment of lipid dysfunction [27]. Additionally, previous reviews and experimental studies reported that lipid metabolism dysfunction may induce age acceleration and play a role in lifespan regulation [28, 29]. Therefore, lipids may act as mediators in the relationship between PA/SB and longevity or age acceleration. However, the mediating roles of lipids in the associations between PA/SB and these outcomes have not been quantified in the literature to date.

Therefore, in this study, we triangulated observational and MR studies to investigate the associations and causations of PA and SB with longevity and AA. Moreover, to explore potential mediating effects of lipids, we further conducted mediation analyses using data from the Guangzhou Biobank Cohort Study (GBCS).

## Methods

### Study design

In this study, we used observational and MR studies to assess the associations and causations of PA and SB with longevity and AA. Using data from the GBCS, we conducted prospective analyses to examine the associations of total PA and PA of different intensities, as well as SB, with longevity. Additionally, we performed cross-sectional analyses to assess the associations with AA. Mediation analyses were employed to identify the mediating roles of lipids in these associations. Furthermore, we conducted two-sample MR studies, with the associations between the genetic variants and exposure and between the variants and outcome estimated from two sets of individuals, to confirm the causal associations of PA and SB with longevity and AA [30].

### Study sample

All participants of GBCS were recruited from September 2003 to January 2008, with baseline characteristics being collected by face-to-face interview using computer-assisted validated questionnaire and detailed physical examination by trained nurses. Details of GBCS have been reported previously [31]. Briefly, GBCS is a 3-way collaboration among the Guangzhou Twelfth People’s Hospital and the Universities of Hong Kong, China, and Birmingham, UK. Participants were recruited from Guangzhou Health and Happiness Association for the Respectable Elders, a community social and welfare organization. Membership is open to Guangzhou permanent residents aged 50 years or above. The Guangzhou Medical Ethics Committee of the Chinese Medical Association approved the study, and all participants gave written, informed consent before participation. In the main analysis of this study, after excluding those with missing information on PA and those lost to follow-up with unknown vital status, the rest of participants that could be classified into longevity and control groups were involved (Figure S1).

### Exposures

The exposure variables were PA and SB, which were assessed by the Chinese Version of the International Physical Activity Questionnaire (IPAQ-C) that has been validated for Chinese adults [32]. Information on the frequencies and durations of walking, moderate, and vigorous activities lasting at least 10 min, and time spent in SB (sitting and lying awake) were assessed. The reported minutes per week in each type of activity were weighted by a metabolic equivalent of the task (MET) based on its energy expenditure, with 1.0 MET for sitting, 3.3 METs for walking, 4.0 METs for MPA, and 8.0 METs for VPA. The data were then converted to metabolic equivalent scores (MET-min/week). PA levels were classified into three categories in ascending order, i.e., low, moderate, and high. Classification criteria for PA levels was shown in the IPAQ guidelines [33]. Moreover, frequency (day/week) and duration (hour/day) specifically for walking, MPA, and VPA, as well as time spent in SB (hour/day) were also included in our analysis.

### Outcomes

For the prospective analyses, the outcome was longevity. Based on a meta-analysis of longevity genome-wide association study (GWAS) [34], in GBCS, longevity cases were defined as participants whose age was at or above the 90th age percentile of the whole GBCS sample (i.e., men: 87.7 years; women: 86.7 years), while controls were participants whose age was at or below the 60th age percentile of the whole GBCS sample (i.e., men: 80.8 years; women: 78.1 years). Age percentile was calculated by sex based on age at the last follow-up (July 2022) or age at the year of death. Participants not classified into the longevity and control groups were excluded. Information on deaths up to July 2022 was obtained via record linkage with the Death Registry of the Guangzhou Centre for Disease Control and Prevention.

We also assessed the cross-sectional associations of PA and SB with AA at baseline. Details of AA were described in our previous study [35]. Briefly, AA was defined as the residual resulting from a linear model that regressed phenotypic age against chronological age, expressed in years. Phenotypic age was calculated based on a weighted linear combination score including chronological age and eight biomarkers (i.e., albumin, creatinine, glucose, C-reactive protein, lymphocyte percent, mean cell volume, red cell distribution width, and white blood cell count). A positive AA value indicated an older biological profile than expected, given the chronological age, whereas a negative value indicated a younger biological profile. AA was also dichotomized into the presence or absence of AA exceeding 5 years.

### Potential confounders and mediators

Potential confounders such as sex, age, social-economic position (education, occupation, and family income) [36], personal lifestyles (smoking status, and alcohol use) [37, 38], and self-rated health [39] which may influence PA, SB, longevity and AA, were included in the analyses. Since AA was calculated as the residual from a linear model regressing phenotypic age on chronological age, baseline age was not considered a potential confounder in the associations of PA and SB with AA. Additionally, since objective health status may also influence PA, SB, longevity and AA, we additionally adjusted for objective health status in the sensitivity model. The poor objective health status was defined as any of the following conditions: (1) regular use of medication for chronic diseases in the past 30 days, (2) any hospital admission during past 6 months, (3) self-reported CVD history, or (4) self-reported cancer history. Since adequate PA could improve lipid metabolism [27], and lipid metabolism dysfunction could induce age acceleration and play a role in lifespan regulation [28, 29], lipid indicators (high-density lipoprotein cholesterol (HDL-C), low-density lipoprotein cholesterol (LDL-C), triglycerides, and total cholesterol) were considered potential mediators in the pathways from PA to longevity and AA. In GBCS, fasting venous blood samples were collected to measure lipids in the Clinical Laboratory of the Guangzhou Twelfth People’s Hospital using a standardized procedure.

### Genetic associations in two-sample MR

In our two-sample MR studies, moderate-to-vigorous physical activity (MVPA) and leisure screen time (LST) were considered exposures, and the outcomes were longevity and AA. Detailed information about the GWAS used in the MR studies is presented in Supplementary methods and Table S1.

### Statistical analyses

The statistical analysis and presentation were consistent with the CHecklist for statistical Assessment of Medical Papers statement [40]. One-way analysis of variance (ANOVA) or Wilcoxon rank-sum test, and chi-square test were used to compare baseline continuous and categorical variables, respectively, by longevity status. Poisson regression with robust error variance was used to assess the longitudinal associations between PA, SB and longevity, yielding relative risks (RRs) and 95% CIs [41]. Linear and logistic regressions were used to examine the cross-sectional associations of PA and SB with AA and AA exceeding 5 years, yielding βs or odds ratios (ORs), and 95% CIs, respectively. To account for varying effects of PA in specific intensities, analyses were repeated for the frequencies and durations of walking, MPA, and VPA. Restricted cubic spline plot with four knots was used to flexibly model the adjusted associations of PA level (metabolic equivalent scores) with longevity and AA exceeding 5 years, with Wald-type test to evaluate potential non-linearity.

Mediation analyses using the “medeff” package in Stata assessed the proportions of the association mediated through lipid indicators (HDL-C, LDL-C, triglycerides and total cholesterol). Sensitivity analyses were conducted to examine the robustness of our results. First, interactions between PA, SB and potential moderators (sex [8] and BMI [42]) on longevity and AA were investigated, along with subgroup analyses stratifying by these factors. Second, given the possible misclassification for those alive in the control group (i.e., they may live longer and become longevity cases), we removed them from the control group and replicated the analyses. Third, since objective health status may also influence PA, SB, longevity and AA, we additionally adjusted for objective health status in the sensitivity model. Moreover, associations of specific PA levels (i.e., metabolic equivalent scores) with longevity and AA were also examined to assess the robustness of the frequency and duration results. Two-sample MRs were conducted to confirm the causality. Details of MR methods are shown in the Supplementary Methods. All statistical analyses were conducted using Stata 16.0 (STATA Corp LP) and R version 4.2.3 (R Foundation for Statistical Computing). A two-sided P value < 0.05 was considered statistically significant.

## Results

### PA, SB and longevity

Of 30,430 participants in GBCS, 20,924 participants were included in the analysis after excluding those with missing information on PA (N = 45), loss to follow-up for vital status (N = 445) and participants not classified into either longevity or control groups (N = 9016) (Figure S1). During an average follow-up of 15.0 years (standard deviation (SD) = 3.7), 2991 participants were classified into the longevity and 17,933 into the control group, with mean baseline age of 73.3 (SD = 3.6) and 57.7 (SD = 4.9) years, respectively. Table 1 shows that participants in longevity group had higher PA level, lower education and family income, and a lower proportion of current smokers and drinkers, but a higher proportion of non-manual job (all P < 0.001). No significant differences in sex or self-rated health status were found between two groups.

**Table 1:** Baseline characteristics by longevity status on 20,924 participants from Guangzhou Biobank Cohort Study in 2003–2008 and followed up till July 2022

	**All participants**	**Longevity**		**P value**
		**No**	**Yes**	
Number of participants, N (%)	20,924 (100.00)	17,933 (85.71)	2991 (14.29)	-
Age, years, mean (SD)	60.0 (7.2)	57.7 (4.9)	73.3 (3.6)	< 0.001
Sex, %				0.987
Men	27.70	27.70	27.68	
Women	72.30	72.30	72.32	
Education, %				< 0.001
Primary or below	38.06	33.44	65.79	
Middle school	53.67	58.13	26.88	
College or above	8.27	8.42	7.33	
Occupation, %				< 0.001
Manual	60.94	60.57	63.15	
Non-manual	22.74	22.01	27.08	
Others	16.32	17.42	9.77	
Family income, CNY/year, %				< 0.001
< 10,000	4.92	4.01	10.38	
10,000–29,999	31.00	29.87	37.80	
30,000–49,999	23.64	25.41	13.02	
≥ 50,000	19.05	20.97	7.53	
Don’t know	21.38	19.73	31.27	
Smoking status, %				< 0.001
Never	80.95	81.10	80.05	
Former	8.55	7.85	12.74	
Current	10.50	11.05	7.21	
Alcohol use, %				< 0.001
Never	70.26	69.12	77.08	
Former	3.71	3.81	3.13	
Current	26.03	27.07	19.79	
Self-rated health, %				0.235
Good	82.38	82.51	81.60	
Poor	17.62	17.49	18.40	
Physical activity level, %				< 0.001
Low	9.38	10.07	5.25	
Moderate	40.60	39.95	44.50	
High	50.02	49.98	50.25	
Metabolic equivalent scores, MET-min/week, median (IQR)	2919 (2772)	2892 (2772)	3012 (2692)	0.083

N = number, SD = standard deviation, CNY = Chinese yuan, MET = metabolic equivalent of the task, IQR = inter-quartile range

Table 2 shows that, after adjusting for sex, baseline age, occupation, education, family income, smoking status, alcohol use and self-rated health, compared with low PA level, moderate and high PA level were positively associated with longevity (RR (95% CI): 1.56 (1.16, 2.11), 1.66 (1.24, 2.21), respectively). Regarding PA in specific intensities, after additionally adjusting for metabolic equivalent scores, walking duration was negatively associated with longevity (RR (95% CI): 0.97 (0.95, 1.00) per hour increment). Frequency and duration of MPA were positively associated with longevity (RR (95% CI): 1.04 (1.03, 1.05) per day increment and 1.05 (1.02, 1.08) per hour increment, respectively), while VPA showed opposite results (RR (95% CI): 0.97 (0.94, 1.00) per day increment and 0.83 (0.70, 0.98) per hour increment, respectively). The association between MPA or VPA level and longevity showed similar results (Table S2). No association was found between SB and longevity (RR (95% CI): 0.99 (0.96, 1.02) per hour increment). Sensitivity analyses removing those alive in the control group or additionally adjusting for objective health status showed consistent results with those in the main analysis (Tables S3-4). We found a significant interaction between sex and PA on longevity (P for interaction = 0.009), with the positive association between PA and longevity being more pronounced in men than in women (Table S5).

**Table 2:** Associations of physical activity and sedentary behavior with longevity in Guangzhou Biobank Cohort Study in 2003–2008 and followed up till July 2022

	**Longevity cases, N (%)**	**Longevity, RR (95% CI)**		
		**Crude model**	**Model 1 **^**a**^	**Model 2 **^**b**^
Physical activity level				
Low	157 (8.00)	1.00	1.00	1.00
Moderate	1331 (15.67)	1.96 (1.67, 2.29) ^***^	1.96 (1.67, 2.29) ^***^	1.56 (1.16, 2.11) ^*^
High	1503 (14.36)	1.80 (1.53, 2.10) ^***^	1.80 (1.53, 2.10) ^***^	1.66 (1.24, 2.21) ^**^
Physical activity frequency, day/week				
Walking	2991 (14.29)	1.13 (1.10, 1.16) ^***^	1.15 (1.12, 1.18) ^***^	1.05 (1.00, 1.10)
MPA	2991 (14.29)	1.06 (1.05, 1.07) ^***^	1.07 (1.06, 1.09) ^***^	1.04 (1.03, 1.05) ^***^
VPA	2991 (14.29)	0.86 (0.82, 0.90) ^***^	0.85 (0.81, 0.89) ^***^	0.97 (0.94, 1.00) ^*^
Physical activity duration, hour/day				
Walking	2991 (14.29)	0.99 (0.97, 1.00)	0.98 (0.95, 1.00) ^*^	0.97 (0.95, 1.00) ^*^
MPA	2991 (14.29)	0.94 (0.90, 0.99) ^*^	0.92 (0.87, 0.98) ^**^	1.05 (1.02, 1.08) ^**^
VPA	2991 (14.29)	0.43 (0.32, 0.58) ^***^	0.41 (0.31, 0.55) ^***^	0.83 (0.70, 0.98) ^*^
Sedentary behavior, hour/day				
Per hour increment	2967 (14.23)	1.03 (1.01, 1.05) ^**^	1.03 (1.01, 1.05) ^**^	0.99 (0.96, 1.02)

N = number, RR = relative risk, CI = confidence interval, MPA = moderate physical activity, VPA = vigorous physical activity

^a^ Model 1: adjusted for metabolic equivalent scores except for physical activity level

^b^ Model 2: Model 1 additionally adjusted for sex, baseline age, occupation, education, family income, smoking status, alcohol use, and self-rated health

^*^P < 0.05, ^**^P < 0.01, ^***^P < 0.001

### PA, SB and AA

**Fig. 1:**
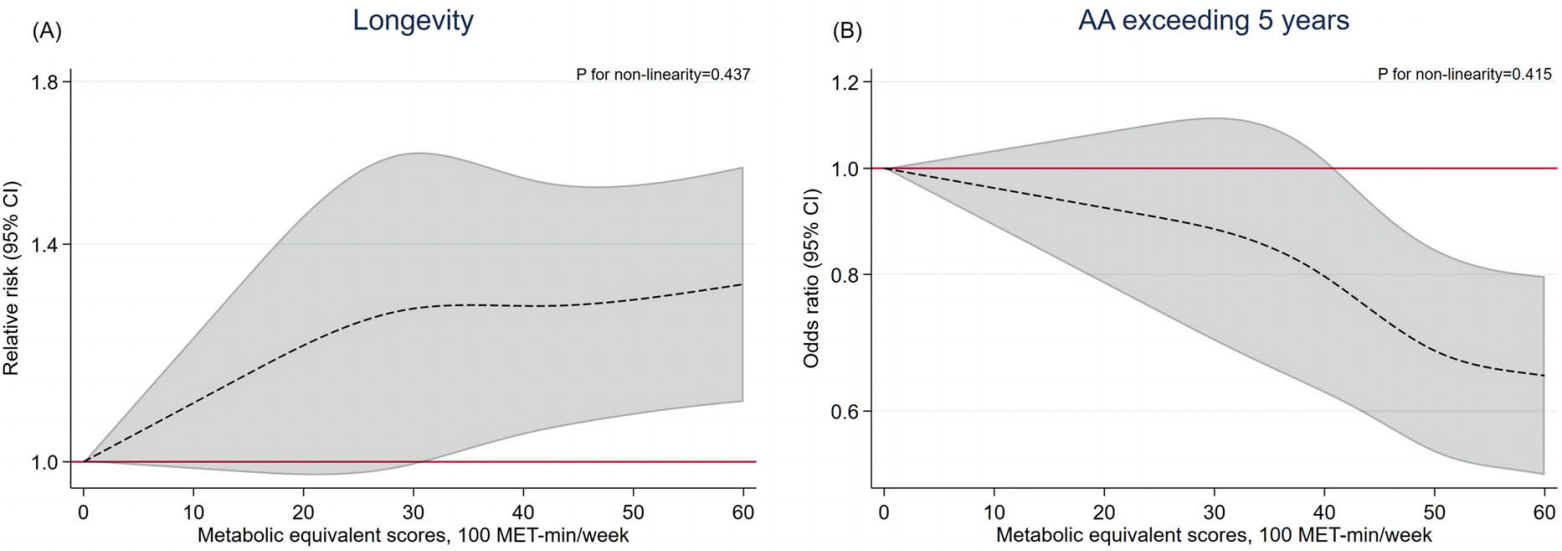
Restricted cubic spline plots for the associations of physical activity level (metabolic equivalent scores) with longevity and age acceleration (AA) exceeding 5 years in Guangzhou Biobank Cohort Study**.** (A) Restricted cubic spline plot for the association of physical activity level (metabolic equivalent scores) with longevity; (B) Restricted cubic spline plot for the association of physical activity level (metabolic equivalent scores) with AA exceeding 5 years. The relative risk/odds ratio and 95% CI above were adjusted for sex, baseline age (for longevity only), occupation, education, family income, smoking status, alcohol use, and self-rated health. AA = age acceleration, MET = metabolic equivalent of the task, CI = confidence interval

In the baseline GBCS from 2003–2004, 9816 participants with AA information were included in this analysis. After similar adjustment, compared with low PA level, moderate and high PA level were associated with lower AA (β (95% CI): -1.43 (-2.41, -0.45) and -2.09 (-3.06, -1.11) years). However, PA in specific intensities, including walking, MPA and VPA were not significantly associated with AA after adjusting for potential confounders (Table 3). Higher MPA frequency was associated with a lower risk of AA exceeding 5 years (OR (95% CI): 0.98 (0.96, 0.99) per day increment), while higher VPA duration was associated with a higher risk of AA exceeding 5 years (OR (95% CI): 1.24 (1.01, 1.53) per hour increment). Walking or MPA level showed negative associations with AA (β (95% CI): -0.02 (-0.03, -0.01) years per 100 MET-min/week increment) or AA exceeding 5 years (OR (95% CI): 0.99 (0.99, 1.00) per 100 MET-min/week increment) (Table S2). Sensitivity model additionally adjusting for objective health status showed consistent results with those in the main analysis (Table S4), No significant interaction was found between sex, BMI, and PA or SB for AA (Table S5). Figure 1 shows that a higher PA level was associated with higher likelihood of longevity and lower likelihood of AA exceeding 5 years, with no evidence of non-linearity (P for non-linearity: 0.437 and 0.415).

**Table 3:** Associations of physical activity and sedentary behavior with age acceleration (AA) and AA exceeding 5 years in Guangzhou Biobank Cohort Study in 2003–2004

	**AA, years, β (95% CI)**			**AA exceeding 5 years, OR (95% CI)**			
	**Crude model**	**Model 1 **^**a**^	**Model 2 **^**b**^	**Cases,** **N (%) **^**c**^	**Crude model**	**Model 1 **^**a**^	**Model 2 **^**b**^
Physical activity level							
Low	0.00	0.00	0.00	37 (26.24)	1.00	1.00	1.00
Moderate	-1.65(-2.67, -0.64) ^**^	-1.65 (-2.67, -0.64) ^**^	-1.43(-2.41, -0.45)^**^	638 (18.58)	0.64 (0.44, 0.94) ^*^	0.64 (0.44, 0.94) ^*^	0.68 (0.45, 1.01)
High	-2.59 (-3.60, -1.58)^***^	-2.59 (-3.60, -1.58)^***^	-2.09 (-3.06, -1.11)^***^	869(13.92)	0.45 (0.31, 0.67)^***^	0.45 (0.31, 0.67)^***^	0.52 (0.35, 0.78)^**^
Physical activity frequency, day/week							
Walking	-0.13 (-0.29, 0.02)	-0.04 (-0.19, 0.12)	-0.02 (-0.17, 0.13)	1544 (15.73)	0.96 (0.90, 1.02)	0.99 (0.93, 1.06)	1.00 (0.93, 1.06)
MPA	-0.12 (-0.16, -0.09)^***^	-0.07 (-0.11, -0.03)^***^	-0.03 (-0.06, 0.01)	1544 (15.73)	0.95 (0.94, 0.97) ^***^	0.97(0.95, 0.98)^***^	0.98 (0.96, 0.99)^*^
VPA	0.09 (-0.01, 0.18)	0.20 (0.10, 0.30)^***^	0.04 (-0.06, 0.13)	1544 (15.73)	1.05 (1.01, 1.09)^*^	1.09 (1.05, 1.14)^***^	1.04 (1.00, 1.09)
Physical activity duration, hour/day							
Walking	-0.25 (-0.33, -0.18)^***^	-0.09 (-0.19, 0.00)	-0.08 (-0.17, 0.01)	1544 (15.73)	0.91 (0.88, 0.95) ^***^	0.98 (0.93, 1.02)	0.98 (0.93, 1.03)
MPA	-0.42 (-0.56, -0.28) ^***^	-0.12 (-0.29, 0.04)	0.00 (-0.16, 0.16)	1544 (15.73)	0.82 (0.76, 0.88)^***^	0.92 (0.84, 1.01)	0.97 (0.89, 1.05)
VPA	0.14 (-0.33, 0.62)	0.82 (0.32, 1.31)^**^	0.12 (-0.37, 0.60)	1544 (15.73)	1.18 (0.98, 1.42)	1.50 (1.23, 1.83)^***^	1.24 (1.01, 1.53)^*^
Sedentary behavior, hour/day							
Per hour increment	0.15 (0.09, 0.21)^***^	0.12 (0.06, 0.18)^***^	0.05 (-0.01, 0.11)	1544 (15.79)	1.12 (1.09, 1.15)^***^	1.04 (1.02, 1.07)^**^	1.02 (0.99, 1.05)

AA = age acceleration, N = number, OR = odds ratio, CI = confidence interval, MPA = moderate physical activity, VPA = vigorous physical activity

^a^ Model 1: adjusted for metabolic equivalent score except for physical activity level

^b^ Model 2: Model 1 additionally adjusted for sex, occupation, education, family income, smoking status, alcohol use, and self-rated health

^c^ Cases were defined as participants with AA exceeding 5 years

^*^P < 0.05, ^**^P < 0.01, ^***^P < 0.001

### Mediation analyses

Table 4 shows that the associations of PA with longevity and AA were partially mediated by lipids after similar adjustment. The largest proportion of the total effect of PA on longevity was mediated by LDL-C (8.23%, 95% CI: 3.58–39.61%). No significant mediation was observed through HDL-C, triglycerides or total cholesterol. Regarding the mediation analysis for AA, the proportions of mediation through LDL-C, triglycerides and total cholesterol were 5.13% (3.94–7.30%), 7.81% (5.98–11.17%), and 3.37% (2.59–4.80%), respectively, with no significant mediation was found for HDL-C.

**Table 4:** Associations of physical activity with longevity and age acceleration (AA) with mediation by lipids in Guangzhou Biobank Cohort Study

Mediators	Indirect effect (ACME) estimates (95% CI) ^a^	Direct effect (ADE) estimates (95% CI) ^a^	Total effect estimates (95% CI) ^a^	Proportion via mediation, % (95% CI)
**Longevity**				
HDL-C, mmol/L	0.000001 (-0.000001, 0.000003)	0.0001 (0.00002, 0.0003) ^*^	0.0001 (0.00002, 0.0003) ^*^	0.51 (0.24, 2.18)
LDL-C, mmol/L	0.00001 (0.000002, 0.00001) ^*^	0.0001 (0.000002, 0.0002) ^*^	0.0001 (0.00001, 0.0003) ^*^	8.23 (3.58, 39.61)
Triglycerides, mmol/L	0.000001 (-0.000001, 0.000003)	0.0001 (0.00002, 0.0003) ^*^	0.0001 (0.00002, 0.0003) ^*^	0.58 (0.28, 2.60)
Total cholesterol, mmol/L	-0.000001 (-0.00001, 0.000004)	0.0001 (0.00002, 0.0003) ^*^	0.0001 (0.00002, 0.0003) ^*^	-0.57 (-2.46, -0.28)
**AA**				
HDL-C, mmol/L	-0.0004 (-0.0009, 0.00003)	-0.0198 (-0.0259, -0.0137) ^*^	-0.0202 (-0.0262, -0.0142) ^*^	1.88 (1.44, 2.67)
LDL-C, mmol/L	-0.0010 (-0.0016, -0.0005) ^*^	-0.0190 (-0.0250, -0.0129) ^*^	-0.0200 (-0.0269, -0.0140) ^*^	5.13 (3.94, 7.30)
Triglycerides, mmol/L	-0.0016 (-0.0027, -0.0004) ^*^	-0.0187 (-0.0246, -0.0126) ^*^	-0.0202 (-0.0264, -0.0141) ^*^	7.81 (5.98, 11.17)
Total cholesterol, mmol/L	-0.0007 (-0.0011, -0.0003) ^*^	-0.0196 (-0.0256, -0.0135) ^*^	-0.0203 (-0.0262, -0.0142) ^*^	3.37 (2.59, 4.80)

ACME = average causal mediated effect, ADE = average direct effect, HDL-C = high-intensity lipoprotein cholesterol, LDL-C = low-intensity lipoprotein cholesterol, AA = age acceleration, CI = confidence interval

^a^: adjusting for sex, baseline age (for longevity only), occupation, education, family income, smoking status, alcohol use, and self-rated health

^*^P < 0.05

### Two-sample MR

A total of 14 and 106 independent SNPs were used as instruments in the MR analyses for examining the associations of MVPA, LST with longevity, and 15 and 111 SNPs for AA, respectively, each showing a mean F-statistic ranging from 37.6 to 39.5. The flow diagrams for SNP selection and summary information on selected SNPs are shown in Figures S2-3 and Table S6. Table 5 shows no significant association of MVPA or LST with longevity using any method. In terms of AA, a positive association between genetically predicted LST and AA was found across all methods, with β (95% CI) of 1.02 (0.67, 1.36) years in the inverse variance weighted (IVW), 0.87 (0.60, 1.14) years in the weighted median (WM) and 0.79 (0.57, 1.00) years in MR pleiotropy residual sum and outlier test (MR-PRESSO). MR-PRESSO showed a marginally negative association between MVPA and AA, with β (95% CI) of -0.63 (-1.23, -0.04) years (P = 0.059). Except for the association between MVPA and longevity, all other associations showed high heterogeneity, with I^2^ values ranging from 69.10% to 79.20%. MR-Egger intercepts indicated no statistical evidence of directional horizontal pleiotropy (all P > 0.05). Sensitivity analyses after removing potential pleiotropy SNPs or using SNPs with P < 5 × 10^–9^ showed consistent results (Tables S7-8).

**Table 5:** Mendelian randomization (MR) estimates of causality of physical activity and sedentary behavior with longevity and age acceleration (AA)

	MR method	Number of SNPs used	Mean F-statistic	β/OR (95% CI)	P value	Cochran’s Q (I^2^)	MR-Egger intercept (P value)	Outliers from MR-PRESSO
Longevity, OR (95% CI)								
MVPA	IVW	14	37.9	1.26 (0.82, 1.94)	0.299	6.54 (0.00)	-0.013 (0.671)	–
	WM			1.37 (0.77, 2.45)	0.288			
	MR-PRESSO			1.26 (0.93, 1.71)	0.166			
	MR-Egger			2.07 (0.20, 21.69)	0.543			
LST	IVW	106	39.5	1.00 (0.74, 1.34)	0.985	339.93 (69.10%)	-0.021 (0.240)	rs6857
	WM			0.93 (0.73, 1.19)	0.578			
	MR-PRESSO			0.88 (0.73, 1.05)	0.163			
	MR-Egger			2.17 (0.57, 8.23)	0.253			
AA, β (95% CI)								
MVPA	IVW	15	37.6	-0.83 (-1.80, 0.15)	0.097	67.15 (79.20%)	-0.053 (0.376)	rs1691471, rs7613360
	WM			-0.42 (-1.14, 0.30)	0.254			
	MR-PRESSO			-0.63 (-1.23, -0.04)	0.059			
	MR-Egger			1.23 (-3.43, 5.88)	0.605			
LST	IVW	111	39.5	1.02 (0.67, 1.36)	< 0.001	481.12 (77.10%)	-0.040 (0.061)	rs6857, rs7615206
	WM			0.87 (0.60, 1.14)	< 0.001			
	MR-PRESSO			0.79 (0.57, 1.00)	< 0.001			
	MR-Egger			2.48 (0.91 4.04)	0.002			

MVPA = moderate-to-vigorous physical activity, LST = leisure screen time, AA = age acceleration, SNP = single-nucleotide polymorphism, OR = odds ratio, CI = confidence interval, IVW = inverse-variance weighted, WM = weighted median, MR-PRESSO = Mendelian randomization pleiotropy residual sum and outlier, MR-Egger = Mendelian randomization Egger regression

## Discussion

To our knowledge, this is the first study to triangulate observational and MR studies in examining the associations and inferring the causality of PA, SB with longevity and AA. In this large population-based cohort study among Chinese, we found a significant positive association between PA and longevity, and a negative association with AA. SB was marginally associated with higher AA in the GBCS, with MR results further confirming this detrimental impact. Regarding PA in specific intensities, MPA was associated with higher likelihood of longevity and lower AA, whereas VPA showed opposite results in the GBCS. Additionally, our mediation analysis in the GBCS found that LDL-C was a significant mediator in the associations between PA and both longevity and AA, while triglycerides and total cholesterol also mediated the association between PA and AA. This study provides comprehensive insights into the complex interactions among PA, SB, longevity, and AA. By illustrating the varying influences of these factors on healthy longevity and aging, our findings highlight the need for differentiated public health strategies to address both physical activity and sedentary lifestyles in promoting healthy longevity and aging.

In our observational study, a higher level of PA was associated with higher likelihood of longevity, and cubic spline analysis showed no non-linearity in this association. Our restricted cubic spline plots also showed that the significantly beneficial impacts of PA level on longevity and reduced AA were observed when PA levels exceeded approximately 4000 METs-min/week. This threshold is roughly equivalent to engaging in 1.5 h of walking plus 1 h of MPA, such as Tai Chi per day. However, a study based on two US cohorts of 85,346 participants reported a U-shaped relationship between PA level and longevity [6]. This discrepancy may be partly explained by different methods of exposure grouping and varying definitions of longevity. Another factor could be the differing BMI levels and obesity rates between China and the US, the latter having much higher rates [43]. Excessively intense PA might impose undue physical and cardiovascular strain on obese individuals, who tend to be more sedentary and may risk acute cardiovascular events with irregular intense PA [44, 45]. Consequently, they might not derive as much health benefit from high PA levels as those of normal weight [42]. Given the generally high BMI level in the US population, the beneficial effect of high PA levels on longevity might not be as evident in the mentioned above. Notably, we found a significant interaction between sex and PA in relation to longevity, aligning with a previous study indicating sex difference and a more favorable effect of PA on longevity in men [8]. This could be attributed to sex-specific physiological cardiovascular responses and adaptations to PA, which may be influenced by differences in genetic, endocrine, and body composition features [46]. Our MR results showed a consistent yet non-significant association between PA and longevity, potentially due to an insufficient number of SNPs. Further GWAS are needed to identify additional SNPs related to PA, and further MR studies are warranted to substantiate the potential protective effect of PA on longevity.

Cumulative evidence has linked PA and SB with various aging-related outcomes, consistently showing the protective effect of PA and detrimental effect of SB, suggesting that both may play significant roles in the aging process. Currently, AA, an aging indicator that considers biological profiles, given chronological age, is widely used [3]. However, evidence on the associations of PA and SB with phenotypic AA is scarce, and existing observational studies have shown inconsistent results [18, 19]. For example, one US study involving 5288 participants showed a negative association of self-reported MVPA with AA (β for 30 min/day increment (95% CI): -0.35(-0.51, -0.19) years) [19]. Consistent with this study, our observational study showed that PA was negatively associated with AA, and no non-linearity was detected in cubic spline analysis. Our study, based on a larger sample size, a more practical PA measurement (MET versus duration), and use of cubic spline analysis, provided more compelling evidence on the protective role of PA in AA. However, another study based on 52,193 UK Biobank participants showed a U-shaped relationship between tertiles of accelerometer-measured PA and AA (β (95% CI) _T2 VS T1_: -0.62 (-0.69, -0.56) years, β (95% CI) _T3 VS T1_: 0.09 (0.03, 0.16) years) [18]. Of note, in this study, AA was defined as the deviation from the average phenotypic age of the cohort participants for individuals of the same sex and age. Hence, different PA measurements and grouping methods, along with varying definitions of AA, may partly account for the discrepancies between this and the other studies. No previous MR study has explored the causal association between PA and AA. Our MR results first detected the negative effect of PA on AA, though the estimates were non-significant but marginal in MR-PRESSO, which may possibly due to a limited number of SNPs. As for SB and AA, consistent with previous findings [19], our observational study showed a positive association between SB and AA. Additionally, our MR study is the first to corroborate the detrimental effect of SB on AA, thereby filling a gap in the current evidence.

Aging is a complex process involving multiple mechanisms [47]. Several molecular mechanisms could explain the impacts of PA and SB on aging. These include the influence on inflammation, oxidative stress, growth factor expression and neural plasticity [48], which may affect maximal aerobic capacity, muscle strength, and metabolic function at the individual level, thereby impacting aging [49]. Our mediation analysis showed that the associations of PA with longevity and AA were mediated by lipids, supporting the metabolic pathways from PA to improve lifespan and delay aging.

We found that PA in different intensities had varying associations with longevity and AA. While MPA showed beneficial effects on both longevity and AA, VPA showed opposite results. VPA has been reported to increase the risk of musculoskeletal complications and CVD, especially in susceptible populations [44]. Given that older adults often have poor physical performance and a high risk of CVD [20, 21], strenuous exercise may not be beneficial for the elderly. In addition, prevalent malnutrition among the elderly may hinder their ability to sustain VPA due to inadequate energy reserves [22]. Notably, a negative association was found between walking duration and longevity. This could be because prolonged walking duration might reflect a slowed walking pace, an indicator of frailty [50], potentially leading to the negative association between walking duration and longevity. In addition, we found an inverse association between MPA duration and longevity in crude model, but a positive association after adjusting for potential confounders. This could be due to the presence of negative confounders (i.e., smoking status and alcohol use). Current smokers and drinkers had longer MPA duration but lower likelihood of longevity. Ignoring these negative confounders could lead to an underestimation of the true association between MPA duration and longevity.

The strengths of our paper included the combination of observational and MR studies to corroborate the causality, the use of mediation analysis to uncover underlying mechanisms, and using detailed exposures, and practical definitions of longevity and age acceleration. Our study also had some limitations. In the observational study, first, PA and SB data were collected via self-reported questionnaire, which are subject to measurement error despite the validation of IPAQ-C in a Chinese population [32]. Second, the relatively small sample size in the low PA level group might lead to underpowered estimates. Third, AA assessment was only conducted at the GBCS baseline, longitudinal analyses on the associations between PA, SB and AA were unavailable. Fourth, we did not distinguish between PA during work and leisure time in our questionnaire, limiting our ability to separately explore the impacts of PA in these contexts. However, given that GBCS participants were individuals aged 50 + years, most of whom were already retired, PA during work periods may not be a major consideration in this study. Regarding the potential limitations of the MR analyses, first, since the GWAS used in our MR study partly included data from UKB [51, 52], sample overlap is a concern, though minimal bias is expected due to valid genetic instruments (i.e., F-statistic > 10) [53] and large biobank source (e.g., UKB) [54]. Second, our MR study showed relatively high heterogeneity. However, we addressed this by using a multiplicative random effect model in the IVW method and various approaches to handle potential pleiotropic effect. Third, the limited numbers of MVPA SNPs might contribute to non-significant results. Moreover, due to the limited number of SNPs associated with specific PA, MR analyses of specific PA with longevity and AA were not conducted, considering potential weak instrument bias.

## Conclusions

In conclusion, PA showed protective effects on longevity and AA, with the effects being partly mediated through lipids, while SB had a detrimental impact on AA. MPA was associated with higher likelihood of longevity and lower AA, whereas VPA showed opposite results. Our findings support current advocacy for “sit less and move more” in the context of healthy longevity and aging, and provide additional evidence on the potential detrimental effects of VPA in the elderly. Further MR studies with a sufficient number of SNPs are warranted to confirm the effects of both total and specific PA on longevity and AA.

## Supplementary Information

Below is the link to the electronic supplementary material.

Supplementary file1 (DOCX 424 KB)

## Data Availability

The datasets used and analyzed during the current study are available from the corresponding author on reasonable request.
