# Supplementary material for: Active longevity and aging: dissecting the impacts of physical and sedentary behaviors on longevity and age acceleration
Source: GeroScience. 2024 Sep 4;47(3):3525–38. doi: 10.1007/s11357-024-01329-3 (PMC12181555; doi:10.1007/s11357-024-01329-3)
Supplement: Supplementary file 1 — Supplementary file1 (DOCX 424 KB) [file 11357_2024_1329_MOESM1_ESM.docx]

**Supplementary materials**

**Title: Active longevity and aging: dissecting the impacts of physical and sedentary behaviors on longevity and age acceleration**

**Journal name: GeroScience**

Ting Yu Lu^1,5^, Jiao Wang, PhD^1,5^, Chao Qiang Jiang, MD^2,5^, Ya Li Jin, MPhil^2^, Kar Keung Cheng, PhD^4^, Tai Hing Lam, MD^2,3,5^, Wei Sen Zhang, PhD^2,5*^, Lin Xu, PhD^1,3,4,5*^

^1^ School of Public Health, Sun Yat-sen University, Guangzhou 510080, China

^2^ Guangzhou Twelfth People’s Hospital, Guangzhou 510620, China

^3^ School of Public Health, the University of Hong Kong, Hong Kong

^4^ Institute of Applied Health Research, University of Birmingham, Birmingham B15 2TT, UK

^5^ Greater Bay Area Public Health Research Collaboration, Guangzhou, China

^*^ Joint corresponding authors

Corresponding author (1): Professor L Xu

School of Public Health, Sun Yat-sen University,

74 Zhongshan 2nd Road, Guangzhou, Guangdong Province, China

Tel: (86) 20-87335523

Fax: (86) 20-87330446

Email: xulin27@mail.sysu.edu.cn

Corresponding author (2): Professor WS Zhang

Guangzhou Twelfth People’s Hospital, Guangzhou 510620, China

Tel: (86) 20-38665762

Email: [zwsgzcn@163.com](mailto:zwsgzcn@163.com)

**Contents**

**Supplementary methods**

**Table S1** Study details for the genome-wide association studies used in Mendelian randomization (MR)

**Table S2** Associations of physical activity level in specific intensities (metabolic equivalent scores) with longevity and age acceleration (AA) in Guangzhou Biobank Cohort Study

**Table S3** Associations of physical activity and sedentary behavior with longevity in Guangzhou Biobank Cohort Study in 2003-2008 and followed up till July 2022 (after removing those alive in the control group)

**Table S4** Associations of physical activity and sedentary behavior with longevity and age acceleration (AA) in Guangzhou Biobank Cohort Study after additionally adjusting for objective health status

**Table S5** Associations of physical activity and sedentary behavior with longevity and age acceleration (AA) stratified by selected baseline characteristics in Guangzhou Biobank Cohort Study

**Table S6** Summary information on the exposure instruments used in Mendelian randomization (MR)

**Table S7** Mendelian randomization (MR) estimates of causality of physical activity and sedentary behavior with longevity and age acceleration (AA) (SNP with P<5×10^-9^)

**Table S8** Mendelian randomization (MR) estimates of causality of physical activity and sedentary behavior with longevity and age acceleration (AA) after removing potential pleiotropy SNPs

**Figure S1** Flow chart of the study sample selection in the observational study in Guangzhou Biobank Cohort Study

**Figure S2** Selection of SNPs for moderate-to-vigorous physical activity related to longevity and age acceleration (AA) used in Mendelian randomization (MR)

**Figure S3** Selection of SNPs for leisure screen time related to longevity and age acceleration (AA) used in Mendelian randomization (MR)

**References**

**Supplementary methods**

***Two-sample Mendelian randomization (MR)***

*Genetic predicted physical activity (PA) and sedentary behavior (SB)*

Genetic associations with moderate-to-vigorous physical activity (MVPA) and leisure screen time (LST) were obtained from the latest publicly available genome-wide association study (GWAS), a meta-analysis of 51 studies with 606,820 and 526,725 individuals of European ancestry for MVPA and LST, respectively [1]. Briefly, MVPA and LST were assessed by self-reported questionnaire. MVPA was categorized as a binary variable (active or inactive) after harmonizing the phenotype across all the studies. LST, assessed in hours per day, included activities such as watching television, playing video games, and computer use. We obtained aggregated data of genetic variants from this GWAS, and extracted single-nucleotide polymorphisms (SNPs) strongly (P<5×10^-8^) and independently (r^2^<0.001) associated with MVPA and LST as instrumental genetic variables.

*Genetic associations with longevity and age acceleration (AA)*

Summary genetic associations with longevity were obtained from a GWAS meta-analysis involving 36,745 individuals of European ancestry from 20 cohorts [2]. The study included 11,262 longevity cases (individuals survived the age at or above the 90th survival percentile) and 25,483 controls (those died or at last follow-up survey at or below the 60th survival percentile). Comprehensive summary statistics of AA was obtained from a GWAS including 107,460 European-descent individuals from UK Biobank (UKB) [3]. In this GWAS, AA was defined as residual from a linear model regressing phenotypic age (derived from chronological age and nine biomarkers) on chronological age. Detailed information about the GWAS used in the MR studies is presented in **Table S1**.

*Statistical analyses*

We used two-sample MR to examine the causality of MVPA, LST with longevity and AA. First, we obtained SNPs strongly (P<5×10^-8^) associated with exposures in each MR. Second, linkage disequilibrium (LD) between SNPs was identified and correlated SNPs were excluded using the “clump_data” function (European population) of “TwoSampleMR” package (r^2^<0.001). Third, we aligned the effect alleles of outcome-associated SNPs to be consistent with the effect alleles of exposure-associated SNPs based on allele letter and allele frequency using “harmonise_data” function. For palindromic SNPs, the effect alleles were confirmed by the relevant available information and then aligned, whereas those that could not be unequivocally aligned were removed. The inverse variance weighted (IVW) with multiplicative random effects, was used as the main analysis by meta-analyzing the SNP-specific Wald estimates. We calculated the F-statistic for each SNP by the square of SNP-exposure association divided by its variance [4, 5], and used mean F-statistic to assess instrument strength. Cochran's Q test and I^2^ statistic were used to assess possible heterogeneity. We also obtained MR estimates using different methods, including the weighted median (WM), MR-Egger regression, and MR pleiotropy residual sum and outlier test (MR-PRESSO). The WM assumes that ≥50% of information from the genetic instruments is valid. A non-zero MR-Egger intercept indicates the presence of horizontal pleiotropy. MR-PRESSO can identify horizontal pleiotropic outliers and correct for pleiotropy via outlier removal, if applicable. As sensitivity analyses, we replicated the MR analysis using SNPs reported in the original GWAS article (P<5×10^-9^) to examine the robustness of our results. “MendelianRandomization”, “MR-PRESSO”, and “TwoSampleMR” packages in R were used to conduct two-sample MR. Our MR study was reported according to the STROBE-MR checklist [6]. Data used in our MR are publicly available summary data, and do not require ethical approval.

***Three key MR assumptions***

MR needs to satisfy three assumptions. First, the genetic instruments should be closely related to the exposure (relevance). We used genome-wide significant (P value<5×10^-8^) independent genetic predictors of the exposures from the latest MVPA and LST GWAS [1]. We calculated the F-statistic to assess the instrument strength, with a value greater than 10 indicating that the instrument is unlikely to be weak. Second, the genetic exposure SNP should not be linked to confounders of the exposure-outcome relation (independence). We checked the associations of selected SNPs with potential confounders identified by previous studies, such as education [7], and lifestyle factors (alcohol intake and smoking status) [8, 9], by comprehensive curated genotype to phenotype cross-references, PhenoScanner V2 [10]. Third, the SNPs that predict the exposures should be associated with the outcomes only via the exposures (exclusion-restriction). Known potentially pleiotropic effects of the selected SNPs, such as obesity indicators (body mass index (BMI), waist circumference, hip circumference, and body fat percentage), and chronic disease (type 2 diabetes, hypertension, high cholesterol, and coronary heart disease), were checked by PhenoScanner V2 [10]. We conducted sensitivity analyses by removing pleiotropic variants, if any. Then, we used MR-Egger intercept and MR-PRESSO to identify statistically any unknown pleiotropic effects [11].

**Table S1 Study details for the genome-wide association studies used in Mendelian randomization (MR)**

| **Phenotype** | **Definition** | **Year** | **Consortium** | **Unit** | **Sample size** | **Adjustment** | **PMID** |
| --- | --- | --- | --- | --- | --- | --- | --- |
| MVPA (exposure) | Self-reported moderate-to-vigorously intensity physical activity during leisure time. | 2022 | 51 European cohorts including Twingen, EPIC-Norfolk, Fenland-OMICS, etc. [1] | - | 606,820 | Sex, age, age^2^, principal components reflecting population structure, and study-specific covariates. | 36071172 |
| LST (exposure) | Self-reported leisure screen time. | 2022 | 51 European cohorts including Twingen, EPIC-Norfolk, Fenland-OMICS, etc. [1] | Hour/day | 526,725 | Sex, age, age^2^, principal components reflecting population structure, and study-specific covariates. | 36071172 |
| Longevity (outcome) | Cases: individuals survived the age at or above the 90th survival percentile. Controls: those died or at last follow-up contract at or below the 60th survival percentile. | 2019 | 20 European cohorts including FHS, Newcastle 85+, etc. [2] | - | 36,745 | Clinical site, known family  relationships, and/or the first four principal components (if applicable). | 31413261 |
| AA (outcome) | The residual resulting from a linear model when regressing Phenotypic Age (a function of chronological age and nine biomarkers) on chronological age. | 2021 | UK Biobank [3] | Year | 107,460 | Chronological age, sex, genotyping array type, and assessment center, random polygenic and environment effects. | 34038024 |

Abbreviations: AA=age acceleration, EPIC-Norfolk=European Prospective Investigation into Cancer Norfolk study, FHS=Framingham Heart Study, GWAS=genome-wide association study, LST=leisure screen time, MVPA=moderate-to-vigorous physical activity.

**Table S2 Associations of physical activity level in specific intensities (metabolic equivalent scores) with longevity and age acceleration (AA) in Guangzhou Biobank Cohort Study**

|  | **Crude model** | **Model 1 ^a^** | **Model 2 ^b^** |
| --- | --- | --- | --- |
| **Longevity, RR (95% CI)** | | | |
| Walking, per 100 MET-min/week | 1.00 (1.00, 1.01) ^***^ | 1.00 (0.99, 1.01) | 1.00 (0.99, 1.01) |
| MPA, per 100 MET-min/week | 1.001 (0.998, 1.004) | 1.01 (1.005, 1.01) ^***^ | 1.01 (1.005, 1.01) ^***^ |
| VPA, per 100 MET-min/week | 0.97 (0.96, 0.98) ^***^ | 1.00 (0.99, 1.00) | 1.00 (0.99, 1.00) |
| **AA, β (95% CI)** | | | |
| Walking, per 100 MET-min/week | -0.03 (-0.04, -0.02) ^***^ | -0.02 (-0.03, -0.01) ^***^ | -0.02 (-0.03, -0.01) ^***^ |
| MPA, per 100 MET-min/week | -0.04 (-0.05, -0.03) ^***^ | -0.02 (-0.03, -0.01) ^***^ | -0.02 (-0.03, -0.01) ^***^ |
| VPA, per 100 MET-min/week | 0.01 (-0.01, 0.03) | -0.01 (-0.03, 0.01) | -0.01 (-0.03, 0.01) |
| **AA exceeding 5 years, OR (95% CI)** | | | |
| Walking, per 100 MET-min/week | 0.99 (0.98, 0.99) ^***^ | 0.99 (0.99, 1.00) ^***^ | 0.99 (0.99, 1.00) ^***^ |
| MPA, per 100 MET-min/week | 0.98 (0.98, 0.99) ^***^ | 0.99 (0.99, 1.00) ^***^ | 0.99 (0.99, 1.00) ^***^ |
| VPA, per 100 MET-min/week | 1.01 (0.99, 1.01) | 1.00 (0.99, 1.01) | 1.00 (0.99, 1.01) |

^a^ Model 1: adjusted for sex, baseline age (for longevity only), occupation, education, family income, smoking status, alcohol use, and self-rated health.

^b^ Model 2: Model 1 additionally mutually adjusted for walking, MPA, and VPA levels, as appropriate.

^*^P<0.05, ^**^P<0.01, ^***^P<0.001.

Abbreviations: AA=age acceleration, CI=confidence interval, MET=metabolic equivalent of the task, MPA=moderate physical activity, OR=odds ratio, RR=relative risk, VPA=vigorous physical activity.

**Table S3 Associations of physical activity and sedentary behavior with longevity in Guangzhou Biobank Cohort Study in 2003-2008 and followed up till July 2022 (after removing those alive in the control group)**

|  | **Longevity cases, N (%)** | **Longevity, RR (95% CI)** | | |
| --- | --- | --- | --- | --- |
|  |  | **Crude model** | **Model 1 ^a^** | **Model 2 ^b^** |
| **Physical activity level** | | | | |
| Low | 157 (36.34) | 1.00 | 1.00 | 1.00 |
| Moderate | 1331 (44.49) | 1.22 (1.07, 1.40) ^**^ | 1.22 (1.07, 1.40) ^**^ | 1.23 (1.06, 1.44) ^**^ |
| High | 1503 (46.63) | 1.28 (1.13, 1.46) ^***^ | 1.28 (1.13, 1.46) ^***^ | 1.28 (1.10, 1.49) ^**^ |
| **Physical activity frequency, day/week** | | | | |
| Walking | 2991 (45.00) | 1.04 (1.02, 1.07) ^***^ | 1.04 (1.02, 1.07) ^***^ | 1.02 (0.99, 1.04) |
| MPA | 2991 (45.00) | 1.03 (1.02, 1.04) ^***^ | 1.03 (1.02, 1.04) ^***^ | 1.02 (1.01, 1.03) ^***^ |
| VPA | 2991 (45.00) | 0.91 (0.88, 0.95) ^***^ | 0.91 (0.87, 0.94) ^***^ | 0.98 (0.95, 1.01) |
| **Physical activity duration, hour/day** | | | | |
| Walking | 2991 (45.00) | 1.02 (1.00, 1.04) ^*^ | 1.02 (1.00, 1.04) | 0.99 (0.97, 1.04) |
| MPA | 2991 (45.00) | 1.02 (0.99, 1.05) | 1.01 (0.97, 1.04) | 1.05 (1.03, 1.07) ^***^ |
| VPA | 2991 (45.00) | 0.65 (0.52, 0.80) ^***^ | 0.61 (0.49, 0.76) ^***^ | 0.89 (0.78, 1.02) |
| **Sedentary behavior, hour/day** | | | | |
| Per hour increment | 2967 (44.91) | 1.01 (0.99, 1.02) | 1.01 (0.99, 1.02) | 0.99 (0.98, 1.01) |

^a^ Model 1: adjusted for metabolic equivalent scores except for physical activity level.

^b^ Model 2: Model 1 additionally adjusted for sex, baseline age, occupation, education, family income, smoking status, alcohol use, and self-rated health.

^*^P<0.05, ^**^P<0.01, ^***^P<0.001.

Abbreviations: CI=confidence interval, MPA=moderate physical activity, N=number, RR=relative risk, VPA=vigorous physical activity.

**Table S4 Associations of physical activity and sedentary behavior with longevity and age acceleration (AA) in Guangzhou Biobank Cohort Study after additionally adjusting for objective health status**

|  | **Longevity, RR (95% CI) ^a^** | **AA, β (95% CI) ^a^** | **AA exceeding 5 years, OR (95% CI) ^a^** |
| --- | --- | --- | --- |
| **Physical activity level** | |  |  |
| Low | 1.00 | 0.00 | 1.00 |
| Moderate | 1.55 (1.15, 2.08) ^**^ | -1.41 (-2.38, -0.44) ^**^ | 0.66 (0.44, 0.99) ^*^ |
| High | 1.65 (1.24, 2.19) ^**^ | -1.98 (-2.38, -1.01) ^***^ | 0.53 (0.36, 0.79) ^**^ |
| **Physical activity frequency, day/week** | |  |  |
| Walking | 1.05 (1.00, 1.10) | -0.02 (-0.17, 0.13) | 0.99 (0.92, 1.06) |
| MPA | 1.04 (1.03, 1.05) ^***^ | -0.03 (-0.06, 0.01) | 0.98 (0.96, 1.00) ^*^ |
| VPA | 0.97 (0.94, 1.00) | 0.05 (-0.05, 0.14) | 1.05 (1.00, 1.09) ^*^ |
| **Physical activity duration, hour/day** | |  |  |
| Walking | 0.97 (0.95, 1.00) ^*^ | -0.07 (-0.16. 0.02) | 0.98 (0.93, 1.03) |
| MPA | 1.05 (1.02, 1.08) ^**^ | -0.01 (-0.17, 0.15) | 0.96 (0.88, 1.05) |
| VPA | 0.83 (0.70, 0.98) ^*^ | 0.08 (-0.39, 0.56) | 1.23 (0.99, 1.51) |
| **Sedentary behavior, hour/day** | |  |  |
| Per hour increment | 0.98 (0.96, 1.01) | 0.06 (0.00, 0.11) | 1.02 (0.99, 1.05) |

^a^ adjusted for metabolic equivalent scores (except for physical activity level), sex, baseline age (for longevity only), occupation, education, family income, smoking status, alcohol use, self-rated health, and objective health status.

^*^P<0.05, ^**^P<0.01, ^***^P<0.001.

Abbreviations: AA=age acceleration, CI=confidence interval, MPA=moderate physical activity, OR=odds ratio, RR=relative risk, VPA=vigorous physical activity.

**Table S5** **Associations of physical activity and sedentary behavior with longevity and age acceleration (AA) stratified by selected baseline characteristics in Guangzhou Biobank Cohort Study**

|  | **Physical activity level** | | | | **Sedentary behavior, hour/day** | |
| --- | --- | --- | --- | --- | --- | --- |
|  | **Low** | **Moderate** | **High** | **P for interaction** | **Per hour increment** | **P for interaction** |
| **Longevity, RR (95% CI) ^a^** | | | | | | |
| *Sex* |  |  |  | 0.009 |  | 0.208 |
| Men | 1.00 | 2.46 (1.16, 5.24) ^*^ | 2.64 (1.26, 5.51) ^*^ |  | 0.97 (0.92, 1.02) |  |
| Women | 1.00 | 1.33 (1.03, 1.71) ^*^ | 1.41 (1.10, 1.80) ^**^ |  | 0.99 (0.97, 1.02) |  |
| *BMI, kg/m^2^* |  |  |  | 0.261 |  | 0.971 |
| <24 | 1.00 | 1.55 (1.08, 2.22) ^*^ | 1.73 (1.23, 2.44) ^**^ |  | 0.99 (0.95, 1.03) |  |
| ≥24 | 1.00 | 1.60 (1.07, 2.41) ^*^ | 1.53 (1.02, 2.31) ^*^ |  | 0.98 (0.95, 1.00) |  |
| **AA, β (95% CI) ^a^** | | | | | | |
| *Sex* |  |  |  | 0.358 |  | 0.728 |
| Men | 0.00 | -2.11 (-3.79, -0.43) ^*^ | -2.90 (-4.56, -1.23) ^**^ |  | 0.06 (-0.04, 0.17) |  |
| Women | 0.00 | -1.15 (-2.36, 0.07) | -1.75 (-2.96, -0.55) ^**^ |  | 0.05 (-0.02, 0.12) |  |
| *BMI, kg/m^2^* |  |  |  | 0.108 |  | 0.107 |
| <24 | 0.00 | -1.18 (-2.46, 0.10) | -1.74 (-3.01, -0.47) ^**^ |  | 0.08 (0.00, 0.16) |  |
| ≥24 | 0.00 | -1.69 (-3.18, -0.20) ^*^ | -2.38 (-3.85, -0.90) ^**^ |  | -0.02 (-0.11, 0.07) |  |
| **AA exceeding 5 years, OR (95% CI) ^a^** | | | | | | |
| *Sex* |  |  |  | 0.824 |  | 0.740 |
| Men | 1.00 | 0.71 (0.39, 1.28) | 0.57 (0.32, 1.02) |  | 1.02 (0.98, 1.06) |  |
| Women | 1.00 | 0.61 (0.36, 1.05) | 0.46 (0.27, 0.78) ^**^ |  | 1.02 (0.98, 1.06) |  |
| *BMI, kg/m^2^* |  |  |  | 0.109 |  | 0.469 |
| <24 | 1.00 | 0.53 (0.31, 0.91) ^*^ | 0.43 (0.25, 0.73) ^**^ |  | 1.02 (0.98, 1.06) |  |
| ≥24 | 1.00 | 0.90 (0.49, 1.63) | 0.67 (0.37, 1.23) |  | 1.00 (0.96, 1.04) |  |

^a^ Adjusted for sex, baseline age (for longevity only), occupation, education, family income, smoking status, alcohol use, and self-rated health, as appropriate.

^*^P<0.05, ^**^P<0.01, ^***^P<0.001.

Abbreviations: AA=age acceleration, BMI=body mass index, CI=confidence interval, OR=odds ratio, RR=relative risk.

**Table S6 Summary information on the exposure instruments used in Mendelian randomization (MR)**

| **Phenotype** | **SNP** | **Chromosome** | **Position** | **Effect allele** | **F-statistic** | **Beta** | **SE** | **P value** | **Gene** | **Potential pleiotropy** |
| --- | --- | --- | --- | --- | --- | --- | --- | --- | --- | --- |
| **MVPA** | rs6427178 | 1 | 169095082 | A | 31.1963 | 0.0229 | 0.0041 | 1.71E-08 | KDM4A | Coronary artery disease |
|  | rs12357890 | 10 | 99762693 | A | 30.1160 | 0.0225 | 0.0041 | 4.77E-08 | CRTAC1 | BMI, waist circumference, hip circumference, body fat percentage |
|  | rs568546 | 11 | 107321156 | T | 33.4140 | 0.0237 | 0.0041 | 5.89E-09 | CWF19L2 | Hypertension |
|  | rs1424751 | 11 | 57479732 | C | 31.0046 | -0.0245 | 0.0044 | 2.53E-08 | MED19 |  |
|  | rs1625595 | 11 | 66078129 | T | 44.3057 | -0.0213 | 0.0032 | 1.90E-11 | CD248 |  |
|  | rs385301 | 17 | 19806828 | T | 36.5124 | -0.0284 | 0.0047 | 1.60E-09 | AKAP10 |  |
|  | rs9903845 | 17 | 50291181 | A | 34.6021 | -0.0200 | 0.0034 | 6.05E-09 | snoZ178 |  |
|  | rs1160545 | 2 | 100832269 | T | 36.8834 | 0.0249 | 0.0041 | 1.73E-09 | LINC01104 | Educational attainment (years), BMI, waist circumference, hip circumference, body fat percentage |
|  | rs2668196 | 3 | 165502709 | A | 32.2056 | -0.0227 | 0.0040 | 2.09E-08 | BCHE |  |
|  | rs336620 | 3 | 18628793 | C | 30.5005 | 0.0243 | 0.0044 | 4.05E-08 | SATB1-AS1 |  |
|  | rs7613360 | 3 | 49916710 | T | 34.5856 | -0.0247 | 0.0042 | 2.77E-09 | ACTBP13 | Educational attainment (years), BMI, body fat percentage, waist circumference, hip circumference, alcohol intake |
|  | rs1691471 | 3 | 85011013 | T | 81.4291 | 0.0379 | 0.0042 | 1.73E-19 | CADM2 |  |
|  | rs4865512 | 5 | 50661601 | A | 32.6531 | 0.0240 | 0.0042 | 7.68E-09 | AC116606.1 |  |
|  | rs4352559 | 5 | 60586625 | T | 31.6406 | 0.0180 | 0.0032 | 1.65E-08 | AC010376.1 | Educational attainment (years) |
|  | rs13201721 | 6 | 141799534 | T | 40.6406 | 0.0255 | 0.0040 | 1.83E-10 | AL356739.1 |  |
|  | rs370935521 | 6 | 26770791 | T | 33.4855 | 0.0434 | 0.0075 | 8.25E-09 | ZFP57 |  |
| **LST** | rs10041724 | 5 | 124273520 | T | 32.0253 | 0.0249 | 0.0044 | 1.51E-08 | LMNB1 | BMI |
|  | rs10059100 | 5 | 120100784 | A | 30.3912 | -0.0215 | 0.0039 | 4.68E-08 | NA |  |
|  | rs1017550 | 10 | 63587683 | A | 31.3600 | 0.0196 | 0.0035 | 2.85E-08 | RP11-491H19.1 |  |
|  | rs10189857 | 2 | 60713235 | A | 61.2865 | -0.0274 | 0.0035 | 7.80E-15 | BCL11A | Educational attainment (years) |
|  | rs10222987 | 4 | 185946130 | A | 33.7045 | -0.0209 | 0.0036 | 9.02E-09 | HELT |  |
|  | rs10253861 | 7 | 8110475 | A | 32.0033 | -0.0198 | 0.0035 | 1.48E-08 | GLCCI1 |  |
|  | rs10400776 | 14 | 97326366 | A | 34.6493 | -0.0259 | 0.0044 | 3.45E-09 | VRK1 |  |
|  | rs10765775 | 11 | 95656362 | A | 32.6949 | -0.0223 | 0.0039 | 9.80E-09 | MTMR2 | Educational attainment (years) |
|  | rs10772643 | 12 | 13415288 | T | 38.5601 | -0.0385 | 0.0062 | 5.88E-10 | EMP1 | Educational attainment (years) |
|  | rs10792966 | 11 | 71544105 | C | 33.4140 | 0.0237 | 0.0041 | 1.06E-08 | DEFB108B |  |
|  | rs10889193 | 1 | 61106174 | A | 38.8225 | 0.0243 | 0.0039 | 4.94E-10 | LINC01748 |  |
|  | rs11074658 | 16 | 10308335 | T | 37.5549 | -0.0239 | 0.0039 | 9.21E-10 | RN7SL493P |  |
|  | rs113838095 | 5 | 138419494 | T | 55.7280 | -0.0321 | 0.0043 | 6.88E-14 | SIL1 |  |
|  | rs114590429 | 2 | 166176789 | A | 39.2711 | 0.0376 | 0.0060 | 3.03E-10 | SCN2A |  |
|  | rs11587591 | 1 | 209762875 | A | 31.5325 | 0.0219 | 0.0039 | 2.23E-08 | CAMK1G |  |
|  | rs1188887 | 6 | 139257866 | T | 39.5979 | 0.0258 | 0.0041 | 2.47E-10 | REPS1 |  |
|  | rs11972285 | 7 | 99025591 | A | 47.0204 | 0.0384 | 0.0056 | 5.13E-12 | ATP5J2-PTCD1 | BMI, waist circumference, body fat percentage, smoking |
|  | rs12062845 | 1 | 98342685 | A | 44.7625 | 0.0281 | 0.0042 | 2.41E-11 | DPYD | Waist circumference, body fat percentage |
|  | rs12206846 | 6 | 108238917 | A | 30.5563 | 0.0199 | 0.0036 | 2.10E-08 | SEC63 |  |
|  | rs12214364 | 6 | 67556372 | T | 29.9452 | -0.0197 | 0.0036 | 4.87E-08 | RNU7-66P |  |
|  | rs12324720 | 15 | 64092140 | A | 34.7075 | -0.0271 | 0.0046 | 3.53E-09 | HERC1 |  |
|  | rs12425850 | 12 | 123501972 | T | 36.6180 | -0.0236 | 0.0039 | 1.37E-09 | PITPNM2 | Educational attainment (years) |
|  | rs12463321 | 19 | 37651855 | A | 33.2195 | -0.0317 | 0.0055 | 6.99E-09 | ZNF585A |  |
|  | rs12617870 | 2 | 193746283 | T | 56.4645 | 0.0263 | 0.0035 | 6.62E-14 | PCGEM1 |  |
|  | rs12678836 | 8 | 92690148 | A | 43.9380 | 0.0232 | 0.0035 | 5.37E-11 | RP11-122C21.1 | Smoking |
|  | rs12962050 | 18 | 35179808 | A | 42.2500 | -0.0234 | 0.0036 | 1.18E-10 | CELF4 | Educational attainment (years) |
|  | rs12981974 | 19 | 19388071 | C | 33.7601 | 0.0337 | 0.0058 | 8.57E-09 | SUGP1 |  |
|  | rs13017586 | 2 | 147847198 | A | 55.1444 | -0.0401 | 0.0054 | 8.25E-14 | AC062032.1 |  |
|  | rs13089152 | 3 | 84765574 | T | 32.5735 | -0.0234 | 0.0041 | 1.53E-08 | LINC00971 |  |
|  | rs13107325 | 4 | 103188709 | T | 36.3591 | 0.0404 | 0.0067 | 1.79E-09 | SLC39A8 | BMI, body fat percentage, hip circumference, alcohol intake, waist circumference, hypertension |
|  | rs13188731 | 5 | 7387097 | A | 29.7025 | 0.0218 | 0.0040 | 4.54E-08 | LINC02142 |  |
|  | rs13235840 | 7 | 133505091 | A | 39.1876 | -0.0313 | 0.0050 | 3.48E-10 | EXOC4 |  |
|  | rs13301354 | 9 | 139924637 | T | 32.5208 | -0.0211 | 0.0037 | 1.22E-08 | FUT7 |  |
|  | rs1362910 | 8 | 30856464 | A | 52.2522 | 0.0253 | 0.0035 | 1.03E-12 | PURG | Waist circumference, body fat percentage, BMI |
|  | rs1375561 | 3 | 85658230 | T | 39.3163 | 0.0232 | 0.0037 | 2.55E-10 | CADM2 | BMI, hip circumference, waist circumference, smoking, body fat percentage, educational attainment (years) |
|  | rs1391954 | 11 | 88575965 | T | 37.5156 | 0.0245 | 0.0040 | 1.51E-09 | GRM5 |  |
|  | rs1445979 | 5 | 60744339 | A | 29.6420 | 0.0196 | 0.0036 | 3.74E-08 | ZSWIM6 |  |
|  | rs16896229 | 4 | 18002583 | T | 34.6021 | 0.0300 | 0.0051 | 3.80E-09 | LCORL |  |
|  | rs1736523 | 1 | 3079885 | C | 30.6746 | 0.0216 | 0.0039 | 2.75E-08 | PRDM16 |  |
|  | rs17621391 | 7 | 140176596 | T | 35.1273 | 0.0243 | 0.0041 | 2.11E-09 | MKRN1 |  |
|  | rs17801257 | 20 | 58892520 | A | 30.3539 | -0.0292 | 0.0053 | 2.92E-08 | MIR646HG |  |
|  | rs1802669 | 10 | 21827796 | A | 87.7192 | 0.0384 | 0.0041 | 2.39E-21 | MLLT10 | Waist circumference, body fat percentage, BMI, hip circumference, |
|  | rs1860337 | 17 | 60851559 | T | 42.0835 | -0.0253 | 0.0039 | 9.08E-11 | MARCH10 |  |
|  | rs1947066 | 5 | 161101615 | A | 45.8698 | 0.0298 | 0.0044 | 8.54E-12 | GABRA6 |  |
|  | rs197439 | 1 | 112280990 | A | 44.1032 | -0.0259 | 0.0039 | 3.29E-11 | FAM212B | BMI, waist circumference, hip circumference |
|  | rs1999065 | 9 | 120514574 | T | 45.2893 | 0.0249 | 0.0037 | 1.24E-11 | snoZ13_snr52 |  |
|  | rs2473977 | 6 | 113454213 | A | 29.3403 | 0.0195 | 0.0036 | 4.60E-08 | RP11-282C5.1 |  |
|  | rs249960 | 5 | 96164771 | A | 35.5216 | 0.0298 | 0.0050 | 2.43E-09 | CTD-2260A17.2 |  |
|  | rs2529484 | 7 | 111180444 | C | 37.6860 | 0.0221 | 0.0036 | 1.39E-09 | IMMP2L |  |
|  | rs262890 | 5 | 62930015 | A | 67.0839 | -0.0344 | 0.0042 | 2.06E-16 | AC010376.1 |  |
|  | rs2667382 | 15 | 83545977 | A | 32.9888 | 0.0224 | 0.0039 | 6.70E-09 | HOMER2 |  |
|  | rs2738284 | 2 | 217311609 | A | 33.7655 | -0.0215 | 0.0037 | 7.05E-09 | SMARCAL1 |  |
|  | rs2783992 | 9 | 1722044 | T | 40.5568 | -0.0242 | 0.0038 | 3.36E-10 | RP11-443B9.1 | Educational attainment (years) |
|  | rs28458909 | 9 | 140257189 | T | 38.0626 | 0.0364 | 0.0059 | 5.48E-10 | EXD3 |  |
|  | rs2964252 | 5 | 152067929 | A | 40.3397 | -0.0235 | 0.0037 | 3.16E-10 | LINC01470 |  |
|  | rs34864022 | 9 | 22609110 | A | 38.3447 | -0.0483 | 0.0078 | 4.71E-10 | LINC01239 |  |
|  | rs36079846 | 2 | 215367159 | T | 39.5575 | -0.0239 | 0.0038 | 4.52E-10 | VWC2L | Alcohol intake |
|  | rs364789 | 5 | 77387439 | A | 39.0625 | 0.0275 | 0.0044 | 2.79E-10 | AP3B1 | Hip circumference |
|  | rs3759344 | 12 | 6862646 | A | 53.1488 | 0.0452 | 0.0062 | 4.26E-13 | MLF2 |  |
|  | rs3781412 | 10 | 126715154 | A | 33.3827 | -0.0208 | 0.0036 | 8.38E-09 | CTBP2 |  |
|  | rs3791033 | 1 | 44134077 | T | 65.1761 | 0.0331 | 0.0041 | 3.66E-16 | KDM4A | Educational attainment (years) |
|  | rs396321 | 5 | 112113735 | T | 37.0359 | -0.0213 | 0.0035 | 1.29E-09 | APC |  |
|  | rs421151 | 8 | 73462574 | A | 32.6531 | -0.0360 | 0.0063 | 1.09E-08 | KCNB2 |  |
|  | rs4303732 | 2 | 100830040 | T | 57.3265 | 0.0265 | 0.0035 | 5.37E-14 | LINC01104 | Educational attainment (years), BMI, waist circumference, hip circumference, body fat percentage, educational attainment (years) |
|  | rs4311996 | 10 | 103735978 | A | 30.9592 | -0.0217 | 0.0039 | 3.56E-08 | C10orf76 |  |
|  | rs4416502 | 4 | 77030872 | A | 36.7539 | 0.0291 | 0.0048 | 1.38E-09 | ART3 |  |
|  | rs4460001 | 4 | 130275243 | A | 30.4073 | -0.0193 | 0.0035 | 4.45E-08 | RP11-404I7.1 |  |
|  | rs4483592 | 11 | 65990439 | T | 47.9290 | 0.0360 | 0.0052 | 3.97E-12 | PACS1 |  |
|  | rs469565 | 22 | 29952437 | T | 31.7018 | -0.0259 | 0.0046 | 1.51E-08 | NIPSNAP1 |  |
|  | rs4889530 | 16 | 31065918 | A | 42.0835 | -0.0253 | 0.0039 | 1.32E-10 | RP11-196G11.6 | Hip circumference, BMI, waist circumference, body fat percentage, hypertension |
|  | rs558134 | 6 | 12693454 | T | 39.0625 | -0.0225 | 0.0036 | 5.05E-10 | RP1-130G2.1 |  |
|  | rs56151256 | 15 | 78024806 | A | 41.9551 | 0.0285 | 0.0044 | 1.17E-10 | LINGO1 | BMI, educational attainment (years), body fat percentage |
|  | rs57092155 | 7 | 53856368 | T | 32.5143 | -0.0268 | 0.0047 | 8.80E-09 | LINC01446 |  |
|  | rs58087899 | 1 | 1863026 | A | 30.8025 | -0.0222 | 0.0040 | 4.35E-08 | CFAP74 | BMI, waist circumference |
|  | rs58541850 | 6 | 166165563 | A | 40.5241 | 0.0522 | 0.0082 | 1.72E-10 | PDE10A |  |
|  | rs6010651 | 20 | 62418243 | A | 34.5156 | 0.0235 | 0.0040 | 3.34E-09 | ZBTB46 |  |
|  | rs6073637 | 20 | 43714051 | A | 35.0589 | -0.0225 | 0.0038 | 4.59E-09 | RP11-323C15.1 |  |
|  | rs6102913 | 20 | 41202958 | T | 30.4073 | -0.0193 | 0.0035 | 2.97E-08 | PTPRT |  |
|  | rs61166637 | 4 | 140771814 | C | 31.6733 | 0.0242 | 0.0043 | 1.48E-08 | MAML3 | Educational attainment (years) |
|  | rs61813324 | 1 | 156049877 | T | 29.5783 | 0.0310 | 0.0057 | 4.37E-08 | MEX3A | BMI, waist circumference, body fat percentage, hip circumference |
|  | rs62068672 | 16 | 89647015 | T | 30.0213 | 0.0263 | 0.0048 | 4.09E-08 | CPNE7 |  |
|  | rs62134209 | 2 | 45093457 | A | 34.9528 | 0.0538 | 0.0091 | 3.28E-09 | LINC01833 |  |
|  | rs62151809 | 2 | 104433256 | T | 34.1775 | 0.0228 | 0.0039 | 3.90E-09 | AC013727.1 | BMI, alcohol intake, smoking, body fat percentage |
|  | rs62244886 | 3 | 71587392 | C | 37.5549 | 0.0239 | 0.0039 | 1.24E-09 | FOXP1 | Alcohol intake |
|  | rs6457816 | 6 | 35362848 | T | 34.9788 | -0.0414 | 0.0070 | 3.97E-09 | PPARD |  |
|  | rs6556840 | 5 | 93463902 | A | 30.3988 | 0.0204 | 0.0037 | 4.32E-08 | CTD-2151A2.3 |  |
|  | rs657412 | 13 | 99047250 | T | 32.3722 | -0.0330 | 0.0058 | 1.40E-08 | FARP1 |  |
|  | rs6674314 | 1 | 243920895 | A | 31.2686 | 0.0274 | 0.0049 | 2.30E-08 | AKT3 |  |
|  | rs6685030 | 1 | 171805284 | A | 38.4400 | -0.0217 | 0.0035 | 5.27E-10 | DNM3 |  |
|  | rs6727997 | 2 | 146346285 | A | 31.6026 | -0.0208 | 0.0037 | 2.04E-08 | AC092484.1 |  |
|  | rs68049022 | 10 | 66407019 | T | 42.7934 | 0.0314 | 0.0048 | 6.18E-11 | RPL17P35 |  |
|  | rs6857 | 19 | 45392254 | T | 61.6392 | -0.0369 | 0.0047 | 5.80E-15 | NECTIN2 | Coronary artery disease, waist circumference, BMI, body fat percentage, T2DM, high cholesterol |
|  | rs71658797 | 1 | 77967507 | A | 41.3582 | 0.0373 | 0.0058 | 1.81E-10 | AK5 | Hip circumference, BMI, waist circumference, body fat percentage |
|  | rs72671494 | 8 | 93195457 | T | 38.8396 | -0.0349 | 0.0056 | 5.72E-10 | RP11-777J24.1 |  |
|  | rs73405293 | 12 | 117522917 | A | 32.5322 | -0.0308 | 0.0054 | 1.35E-08 | TESC |  |
|  | rs73420302 | 17 | 77768068 | C | 34.6021 | -0.0300 | 0.0051 | 3.04E-09 | CBX8 |  |
|  | rs7430216 | 3 | 75201030 | T | 35.4308 | 0.0250 | 0.0042 | 2.50E-09 | LINC02050 |  |
|  | rs743699 | 4 | 3305116 | A | 36.8228 | -0.0267 | 0.0044 | 1.18E-09 | RGS12 | BMI |
|  | rs74996610 | 12 | 24075007 | C | 30.4277 | -0.0513 | 0.0093 | 3.12E-08 | SOX5 |  |
|  | rs7615206 | 3 | 49937505 | T | 97.1633 | -0.0345 | 0.0035 | 1.50E-22 | MST1R | BMI, educational attainment (years), waist circumference, body fat percentage, hip circumference, alcohol intake |
|  | rs7616518 | 3 | 83530809 | A | 31.3600 | 0.0196 | 0.0035 | 2.60E-08 | HSPE1P19 |  |
|  | rs76267866 | 3 | 70540347 | A | 39.9316 | -0.0297 | 0.0047 | 3.53E-10 | PROK2 |  |
|  | rs7627290 | 3 | 165711001 | A | 35.0828 | -0.0231 | 0.0039 | 2.36E-09 | SLC2A2 |  |
|  | rs76602404 | 7 | 50737835 | T | 29.4694 | -0.0266 | 0.0049 | 4.38E-08 | GRB10 |  |
|  | rs7821826 | 8 | 10769439 | T | 34.9788 | 0.0207 | 0.0035 | 3.25E-09 | XKR6 | BMI, hypertension |
|  | rs78394231 | 6 | 107649123 | T | 34.3576 | -0.0381 | 0.0065 | 3.53E-09 | PDSS2 |  |
|  | rs78451709 | 18 | 41515058 | T | 33.3025 | -0.0277 | 0.0048 | 9.66E-09 | RNU6-443P |  |
|  | rs7875078 | 9 | 14494845 | A | 33.9722 | 0.0204 | 0.0035 | 7.41E-09 | RP11-408A13.2 |  |
|  | rs7969719 | 12 | 109883577 | T | 53.6457 | 0.0271 | 0.0037 | 4.46E-13 | MYO1H |  |
|  | rs7991062 | 13 | 100713194 | C | 66.7609 | -0.0335 | 0.0041 | 1.32E-16 | ASNSP3 |  |
|  | rs841020 | 10 | 125409953 | T | 37.3765 | 0.0269 | 0.0044 | 1.15E-09 | GPR26 |  |
|  | rs892087 | 19 | 10794793 | T | 50.4100 | -0.0284 | 0.0040 | 9.98E-13 | ILF3 | Hip circumference |
|  | rs9278004 | 6 | 33319815 | A | 47.7796 | 0.0394 | 0.0057 | 3.23E-12 | TBC1D22B | Waist circumference, hip circumference, BMI |
|  | rs9713906 | 3 | 93543006 | A | 34.5156 | -0.0235 | 0.0040 | 3.86E-09 | RNU6-461P | Body fat percentage |
|  | rs9867121 | 3 | 114631548 | A | 40.1956 | -0.0317 | 0.0050 | 2.02E-10 | ZBTB20 |  |

Abbreviations: BMI=body mass index, LST=leisure screen time, MVPA=moderate-to-vigorous physical activity, T2DM=type 2 diabetes mellitus, SNP=single-nucleotide polymorphism, SE=standard error.

**Table S7 Mendelian randomization (MR) estimates of causality of physical activity and sedentary behavior with longevity and age acceleration (AA) (SNP with P<5**×**10^-9^)**

|  | **MR method** | **Number of SNPs used** | **Mean F-statistic** | **β/OR (95% CI)** | **P value** | **Cochran’s Q (I^2^)** | **MR-Egger intercept (P value)** | **Outliers from MR-PRESSO** |
| --- | --- | --- | --- | --- | --- | --- | --- | --- |
| **Longevity, OR (95% CI)** | | | | | | | |  |
| MVPA | IVW | 6 | 45.7 | 1.40 (0.78, 2.55) | 0.262 | 4.34 (0.00) | -0.016 (0.706) | - |
|  | WM |  |  | 1.47 (0.69, 3.14) | 0.314 |  |  |  |
|  | MR-PRESSO |  |  | 1.40 (0.81, 2.44) | 0.282 |  |  |  |
|  | MR-Egger |  |  | 2.52 (0.11, 55.59) | 0.558 |  |  |  |
| LST | IVW | 57 | 43.0 | 1.05 (0.65, 1.70) | 0.838 | 284.69 (80.30%) | -0.047 (0.166) | rs13107325, rs6857 |
|  | WM |  |  | 0.98 (0.71, 1.35) | 0.909 |  |  |  |
|  | MR-PRESSO |  |  | 0.88 (0.69, 1.11) | 0.280 |  |  |  |
|  | MR-Egger |  |  | 5.49 (0.51, 59.68) | 0.162 |  |  |  |
| **AA, β (95% CI)** | | | | | | | |  |
| MVPA | IVW | 6 | 45.7 | -1.15 (-3.24, 0.93) | 0.279 | 54.57 (90.8%) | -0.174 (0.229) | rs1691471, rs7613360 |
|  | WM |  |  | -0.34 (-1.42, 0.74) | 0.535 |  |  |  |
|  | MR-PRESSO |  |  | -0.87 (-2.05, 0.30) | 0.242 |  |  |  |
|  | MR-Egger |  |  | 5.02 (-5.24, 15.29) | 0.337 |  |  |  |
| LST | IVW | 58 | 42.9 | 1.33 (0.76, 1.90) | <0.001 | 382.22 (85.10%) | -0.070 (0.079) | rs6857,  rs7615206 |
|  | WM |  |  | 1.17 (0.81, 1.53) | <0.001 |  |  |  |
|  | MR-PRESSO |  |  | 0.93 (0.63, 1.23) | <0.001 |  |  |  |
|  | MR-Egger |  |  | 3.78 (0.99 6.57) | 0.008 |  |  |  |

Abbreviations: AA=age acceleration, CI=confidence interval, IVW=inverse-variance weighted, LST=leisure screen time, MR-Egger=Mendelian randomization Egger regression, MR-PRESSO=Mendelian randomization pleiotropy residual sum and outlier, MVPA=moderate-to-vigorous physical activity, OR=odds ratio, SNP=single-nucleotide polymorphism, WM=weighted median.

**Table S8 Mendelian randomization (MR) estimates of causality of physical activity and sedentary behavior with longevity and age acceleration (AA) after removing potential pleiotropy SNPs**

|  | **MR method** | **Number of SNPs used** | **Mean F-statistic** | **β/OR (95% CI)** | **P value** | **Cochran’s Q (I^2^)** | **MR-Egger intercept (P value)** | **Outliers from MR-PRESSO** |
| --- | --- | --- | --- | --- | --- | --- | --- | --- |
| **Longevity, OR (95% CI)** | | | | | | | |  |
| MVPA | IVW | 8 | 41.6 | 1.23 (0.71, 2.14) | 0.464 | 2.72 (0.00) | -0.013 (0.705) | - |
|  | WM |  |  | 1.33 (0.66, 2.68) | 0.417 |  |  |  |
|  | MR-PRESSO |  |  | 1.23 (0.87, 1.74) | 0.278 |  |  |  |
|  | MR-Egger |  |  | 2.03 (0.14, 28.56) | 0.600 |  |  |  |
| LST | IVW | 75 | 36.9 | 0.87 (0.70, 1.07) | 0.192 | 86.03 (14.00%) | -0.008 (0.538) | - |
|  | WM |  |  | 0.96 (0.72, 1.29) | 0.804 |  |  |  |
|  | MR-PRESSO |  |  | 0.87 (0.70, 1.07) | 0.196 |  |  |  |
|  | MR-Egger |  |  | 1.15 (0.45, 2.92) | 0.767 |  |  |  |
| **AA, β (95% CI)** | | | | | | | |  |
| MVPA | IVW | 9 | 40.7 | -0.19 (-1.04, 0.66) | 0.659 | 18.31 (56.30%) | -0.040 (0.425) | - |
|  | WM |  |  | 0.30 (-0.52, 1.12) | 0.472 |  |  |  |
|  | MR-PRESSO |  |  | -0.19 (-1.03, 0.66) | 0.671 |  |  |  |
|  | MR-Egger |  |  | 1.25 (-2.40, 4.89) | 0.502 |  |  |  |
| LST | IVW | 77 | 37.1 | 0.62 (0.38, 0.87) | <0.001 | 111.46 (31.80%) | 0.008 (0.572) | - |
|  | WM |  |  | 0.69 (0.37, 1.00) | <0.001 |  |  |  |
|  | MR-PRESSO |  |  | 0.62 (0.37, 0.87) | <0.001 |  |  |  |
|  | MR-Egger |  |  | 0.32 (-0.77 1.40) | 0.566 |  |  |  |

Abbreviations: AA=age acceleration, CI=confidence interval, IVW=inverse-variance weighted, LST=leisure screen time, MR-Egger=Mendelian randomization Egger regression, MR-PRESSO=Mendelian randomization pleiotropy residual sum and outlier, MVPA=moderate-to-vigorous physical activity, OR=odds ratio, SNP=single-nucleotide polymorphism, WM=weighted median.

**Figure S1 Flow chart of the study sample selection in the observational study in Guangzhou Biobank Cohort Study**

GBCS=Guangzhou Biobank Cohort Study, PA=physical activity.

**Figure S2 Selection of SNPs for moderate-to-vigorous physical activity related to longevity and age acceleration (AA) used in Mendelian randomization (MR)**

SNP=single-nucleotide polymorphism, MVPA=moderate-to-vigorous physical activity, AA=age acceleration.

**Figure S3 Selection of SNPs for leisure screen time related to longevity and age acceleration (AA) used in Mendelian randomization (MR)**

SNP=single-nucleotide polymorphism, LST=leisure screen time, AA=age acceleration.

**References**

[1] Wang Z, Emmerich A, Pillon NJ, Moore T, Hemerich D, Cornelis MC*, et al.* (2022). Genome-wide association analyses of physical activity and sedentary behavior provide insights into underlying mechanisms and roles in disease prevention. Nat Genet, 54:1332-1344.

[2] Deelen J, Evans DS, Arking DE, Tesi N, Nygaard M, Liu X*, et al.* (2019). A meta-analysis of genome-wide association studies identifies multiple longevity genes. Nat Commun, 10:3669.

[3] Kuo CL, Pilling LC, Liu Z, Atkins JL, Levine ME (2021). Genetic associations for two biological age measures point to distinct aging phenotypes. Aging Cell, 20:e13376.

[4] Bowden J, Del Greco MF, Minelli C, Davey Smith G, Sheehan NA, Thompson JR (2016). Assessing the suitability of summary data for two-sample Mendelian randomization analyses using MR-Egger regression: the role of the I2 statistic. Int J Epidemiol, 45:1961-1974.

[5] Pierce BL, Ahsan H, Vanderweele TJ (2011). Power and instrument strength requirements for Mendelian randomization studies using multiple genetic variants. Int J Epidemiol, 40:740-752.

[6] Skrivankova VW, Richmond RC, Woolf BAR, Davies NM, Swanson SA, VanderWeele TJ*, et al.* (2021). Strengthening the reporting of observational studies in epidemiology using mendelian randomisation (STROBE-MR): explanation and elaboration. Bmj, 375:n2233.

[7] Sumimoto Y, Yanagita M, Miyamatsu N, Okuda N, Nishi N, Nakamura Y*, et al.* (2021). Association between socioeconomic status and prolonged television viewing time in a general Japanese population: NIPPON DATA2010. Environ Health Prev Med, 26:57.

[8] Salin K, Kankaanpää A, Hirvensalo M, Lounassalo I, Yang X, Magnussen CG*, et al.* (2019). Smoking and Physical Activity Trajectories from Childhood to Midlife. Int J Environ Res Public Health, 16.

[9] Dodge T, Clarke P, Dwan R (2017). The Relationship Between Physical Activity and Alcohol Use Among Adults in the United States. Am J Health Promot, 31:97-108.

[10] Kamat MA, Blackshaw JA, Young R, Surendran P, Burgess S, Danesh J*, et al.* (2019). PhenoScanner V2: an expanded tool for searching human genotype-phenotype associations. Bioinformatics, 35:4851-4853.

[11] Verbanck M, Chen CY, Neale B, Do R (2018). Detection of widespread horizontal pleiotropy in causal relationships inferred from Mendelian randomization between complex traits and diseases. Nat Genet, 50:693-698.
